# Effect of Early Curing Temperature on the Tunnel Fire Resistance of Self-Compacting Concrete Coated with Aerogel Cement Paste

**DOI:** 10.3390/ma14195782

**Published:** 2021-10-03

**Authors:** Kai-Lin Huang, Shu-Jin Li, Ping-Hua Zhu

**Affiliations:** 1 School of Civil and Architectural Engineering, Changzhou Institute of Technology, Changzhou 213032, China; hkailin@yeah.net; 2 Department of Civil Engineering, School of Environmental and Safety Engineering, Changzhou University, Changzhou 213164, China; zph@cczu.edu.cn

**Keywords:** early curing temperature, aerogel cement paste, self-compacting concrete, tunnel fire resistance

## Abstract

In this paper, the effect of early curing temperature on the tunnel fire resistance of self-compacting concrete (SCC) coated with aerogel cement paste (ACP) was studied. The physical properties in terms of the compressive strength, flexural strength, and thermal conductivity of ACP were tested under different early curing temperatures. The tunnel fire resistance of ACP and SCC coated with ACP was determined, and the microstructure of ACP and SCC after a tunnel fire were characterized by scanning electron microscopy. The results show that the strength of ACP initially increased (by 10–40 °C) and then later decreased (by 40–60 °C) with the increase in early curing temperature. ACP under 40 °C early curing exhibited the minimum number of cracks and mass loss after the tunnel fire. Too high or too low early curing temperature reduced the thermal conductivity of ACP but accelerated the formation and expansion of microcracks during the tunnel fire. The residual compressive strength of SCC coated with ACP under 40 °C early curing after the tunnel fire was the highest, demonstrating the best tunnel fire resistance.

## 1. Introduction

Self-compacting concrete (SCC) was first developed for antiseismic reinforced concrete in 1986 in Japan [1]. Due to its high flowability, it can flow and fill the formwork under its own weight without external force [2,3]. SCC shows good performance in terms of segregation resistance, mechanical properties, and durability [4]. Moreover, the application of SCC decreases the construction time and structure cost, representing sustainable development [5]. Because of its excellent properties, SCC is widely used all over the world in many concrete structures, especially in tunnel structure [6,7].

However, the SCC in tunnel structures encounter more challenges in the event of tunnel fires [8]. When a fire occurs, SCC will spall, cracks will generate, and its strength will decline. The main mechanism leading to failure of SCC in a tunnel fire is that the high temperature of the tunnel fire alters the pore structure of the SCC and reduces the compressive strength, leading to failure of the concrete structure [9,10]. Moreover, SCC is more vulnerable to fire attack [11]. Due to the low porosity and permeability, the water vapor pressure is not easy to release; the high internal pressure could cause spalling of the concrete. According to the Chinese code GB 50016-2014, the fire resistance limit of a tunnel load-bearing structure under the standard CH heating curve is 2.5 h. Therefore, it is of significant importance to improve the tunnel fire resistance of SCC. To solve this problem, fire-resistant coating is a useful method to limit the heat transfer in concrete that has recently received increasing attention [12,13].

Aerogel is defined as a type of nanostructured material with high porosity and specific surface area [14]. In recent years, SiO_2_ aerogel has become a research hotspot due to its low thermal conductivity and hydrophobicity, which has been widely used in aerogel insulation boards, aerogel insulation blankets, and other thermal insulation materials [15,16]. There are few studies on the application of SiO_2_ aerogel cement paste (ACP) as a fireproof coating. Kim et al. [17] reduced the thermal conductivity of cement slurry by adding aerogel powder to cement. Liu et al. [18] compared the thermal insulation effect between SiO_2_ aerogel-coated lining and conventional lining after a simulated fire, and the internal temperature of the SiO_2_ aerogel-coated lining was far below that of the conventional lining. Zhu et al. [19] found that using ACP improved the fire safety of high-performance concrete linings in tunnels. Using aerogel mortar as a fireproof coating can significantly improve the heat insulation performance of SCC in tunnel fires.

The curing regime has a great significance on the porosity of ACP, which determines thermal conductivity and tunnel fire resistance. Among many factors, curing temperature is the key factor affecting the hydration degree of ACP. Tam et al. [20] studied the effects of a thermal curing temperature of 100 °C at durations of 8 h, 16 h, and 24 h on concrete microstructure. Results showed that continuous high-temperature curing had a positive effect on the formation of crystal hydrate. Ng et al. [21] proposed that appropriate curing regimes can improve the properties of aerogel-incorporated mortar to achieve the desired requirements. Wang et al. [22] studied the effect of curing temperature on the tunnel fire insulation of the ACP coating. Current research on the fire resistance of ACP mainly focuses on SiO_2_ aerogel content and coating. However, the influence of an early curing method on tunnel fire resistance of SCC coated with ACP is not clear.

Hydration kinetics of cementitious materials are closely related to curing temperature. Appropriate curing temperature can not only improve the fire resistance of ACP but also allow formulating corresponding construction strategies of tunnel fireproof coating to improve production efficiency. Therefore, this paper aims to reveal the effect of early curing temperature on the tunnel fire resistance of SCC coated with ACP. The compressive strength, flexural strength, and thermal conductivity of ACP were tested under different early curing temperatures. The tunnel fire resistance of ACP and SCC coated with ACP was determined and the microstructure of ACP and SCC after a tunnel fire was characterized by scanning electron microscopy (SEM). The research results can provide optimal new theories and methods for the maintenance of ACP and provide a valuable theoretical basis for the revision of ACP technology in the future.

## 2. Experimental

### 2.1. Materials

**Figure 1:**
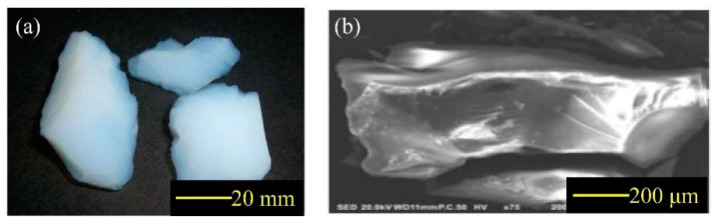
Aerogel: (**a**) macroscopic morphology; (**b**) SEM image.

**Figure 2:**
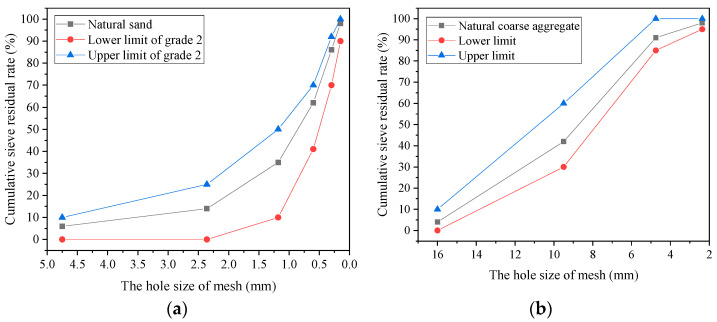
Gradation curve of aggregates: (**a**) natural sand; (**b**) natural coarse aggregate.

Ordinary Portland cement (P.O 42.5), silica fume, fly ash, and slag were used as cementitious materials. The chemical compositions of cementitious materials are shown in Table 1. The air-entraining agent (GYQ-I) and polycarboxylate high-performance water reducer (JK-PCA) were from Jiangsu Subor Co. Ltd., Nanjing City, Jiangsu Province, China. Performance indicators for additives are shown in Table 2 and Table 3. The hydrophobic aerogel granules were provided by Guangdong Alison Hi-Tech Co. Ltd., Yingde City, Guangdong Province, China. The macroscopic morphology and scanning electron microscopy (SEM) pictures of aerogel are shown in Figure 1; the physical properties of aerogel are shown in Table 4. Crushed limestone with a size range of 5–16 mm was used as the natural coarse aggregate. Local river sand was used as the fine aggregate, which had a fineness modulus of 2.4. The gradation curves of aggregates are shown in Figure 2.

### 2.2. Preparation of ACP and SCC

ACP was prepared according to the premixed cement paste process [23]. The specific mix proportion of the ACP that was used was designed as shown in Table 5. The sizes of the ACP samples were 40 mm × 40 mm × 160 mm and 250 mm × 250 mm × 50 mm. The mix proportion of SCC is shown in Table 6. The ACP strength was 40 MPa (C40). The size of the SCC samples was 100 mm × 100 mm × 100 mm. In order to determine the tunnel fire resistance of concrete, one surface of the SCC was coated with ACP; the thickness of the ACP coating was 10 mm. The flow chart of the ACP composite SCC specimen preparation is available in a previously published paper [22].

After casting, all the samples were employed with different curing methods. For the early curing (1 day before demolding), the temperature was set as 10 °C, 20 °C, 30 °C, 40 °C, 50 °C, and 60 °C; the relative humidity was above 95%. The early curing lasted 1 day. After that, it was demolded and experienced 14 days wet curing and 14 days dry curing, simulated with on-site construction curing. The average temperature during the experiment period was 13–24 °C and the relative humidity was 70 ± 15%. For the 14 days wet curing, the surface of the specimen was sprayed with water every 6 h and covered with a film. The different curing regimes are presented in Table 7.

### 2.3. Measurements

#### 2.3.1. Physical and Mechanical Properties of ACP

The flexural strength of the ACP sample (40 mm × 40 mm × 160 mm) was measured with a WA-600C electro-hydraulic servo universal testing machine (Wuxi xinluda Instrument Equipment Co., Ltd., Wuxi city, Jiangsu Province, China), and the final value was taken as the average value of the three specimens. The compressive strength was measured on the six prisms generated by the flexural test. The number of specimens used for determining the ACP physical properties was 36. The final value in this paper was the average of the three test results.

The thermal conductivity of the ACP sample (250 mm × 250 mm × 50 mm) was tested by hot-wire method [24]. For specific methods and principles, see [24]. The thermal conductivity of the ACP can be obtained by Equation (1):

(1) $$\lambda =\frac{Q}{4\pi \left[T(t_{2})-T(t_{1})\right]}\ln(\frac{t_{2}}{t_{1}})$$

where *λ* is thermal conductivity, (W/m·K); *Q* is the power of unit length of the heating source, (W/m); *T* is temperature at time t, (K); and *t* is time, (s).

In order to accurately compare the thermal conductivity of ACP under different curing methods, three specimens were measured, and the average value was taken. The number of specimens used for ACP thermal conductivity was 18.

#### 2.3.2. Simulated Tunnel Fire

A simulated tunnel fire test was conducted on the SCC coated with ACP, ACP samples, and 10 mg ACP powders. An RTD-45-13 Bend resistance furnace (Jiangsu Jinhuan Test Equipment Co., Ltd., Taizhou City, Jiangsu Province, China) was used as the tunnel fire test device. The temperature of the tunnel fire reached 1100 °C within 30 min and maintained for 2.5 h, according to the HC standard tunnel fire temperature rising curve. The resistance furnace and temperature rising curve of the tunnel fire is shown in a previous paper [22]. To ensure that only the ACP surface was tested for the simulation tunnel fire, the uncoated surface of the SCC was covered with a 100 mm thick zirconium fiber blanket. The number of specimens used for the simulated tunnel fire test was 36.

The macroscopic morphology of the ACP before and after the simulated tunnel fire was analyzed. In addition, the mass loss of the ACP samples (50 mm × 50 mm × 50 mm) and 10 mg ACP powder before and after the simulated tunnel fire were measured to assess the fire resistance of the ACP under various curing regimes. The tunnel fire resistance of the SCC coated with ACP was determined in terms of the residual compressive strength.

#### 2.3.3. SEM

Before and after the tunnel fire, samples were taken at the center of the specimen (ACP and SCC coated with ACP) surface. The morphology of samples was analyzed using a Supra 55 scanning electron microscope (Carl Zeiss, Oberkochen, Baden Voorburg, Germany); the SEM image was carried out under 20 kV acceleration voltage.

## 3. Results and Discussion

### 3.1. Physical Properties of ACP

**Figure 3:**
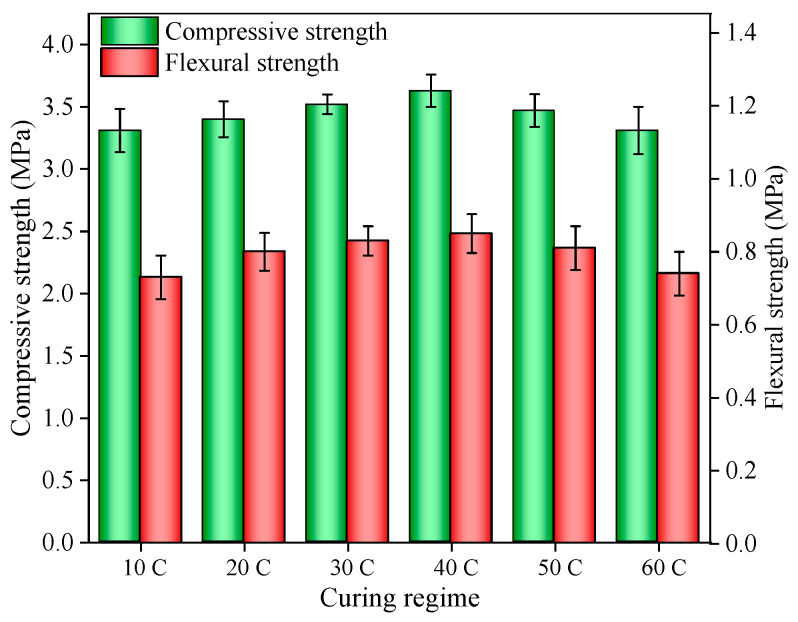
Compressive strength and flexural strength of ACP under different early curing regimes.

The compressive strength and flexural strength of the ACP under different early curing regimes are shown in Figure 3. The compressive strength and flexural strength of ACP increased at first and then decreased later with the increase of early curing temperature. More specifically, the highest compressive strength (3.63 MPa) and flexural strength (0.85 MPa) of ACP occurred in the specimen under 40 °C early curing. The compressive strength and flexural strength of ACP under 10 °C early curing were reduced by 4% and 5%, respectively, compared with that under 40 °C early curing. This suggests that increasing the early curing temperature appropriately (no more than 40 °C) was beneficial in accelerating the hydration reaction and in raising the compressive strength and flexural strength of ACP [25]. Conversely, when the early curing temperature was higher than 40 °C, the compressive strength and flexural strength of ACP gradually decreased with the increase of early curing temperature and reached the lowest point at 60 °C. This was due to the high early curing temperature in the curing process, leading to the formation of internal cracks and a reduction in the mechanical properties of ACP [26]. These internal cracks could reduce the durability and service life of ACP [27].

**Figure 4:**
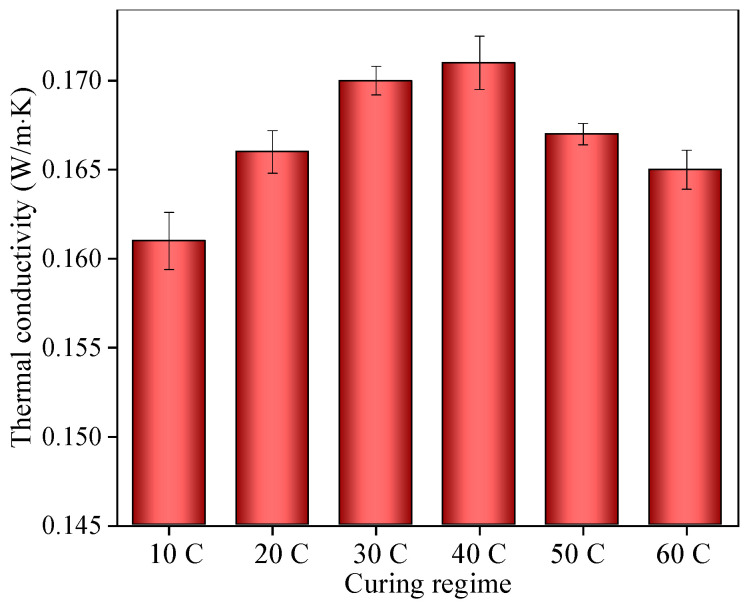
Thermal conductivity of ACP under different early curing regimes.

The thermal conductivity of ACP under different early curing temperatures is shown in Figure 4. It can be seen that the thermal conductivity increased at first as the early curing temperature increased up to 40 °C and then decreased when the temperature increased from 40 °C to 60 °C. Nevertheless, in all cases, ACP had low thermal conductivity and good thermal insulation.

The thermal conductivity reached a maximum value of 0.171 W/(m∙K) in the specimen under 40 °C early curing. The thermal conductivity of ACP under 10 °C early curing was the lowest, nearly 3% less than that under 40 °C early curing. The low early curing temperature slowed the hydration rate and increased the porosity of the ACP, leading to low thermal conductivity. This indicates a better thermal insulation performance of ACP with low early curing. In addition, the tunnel fire resistance could also be reduced due to the large porosity. It is necessary to further analyze the tunnel fire resistance of ACP.

### 3.2. Macroscopic Morphology of ACP

**Figure 5:**
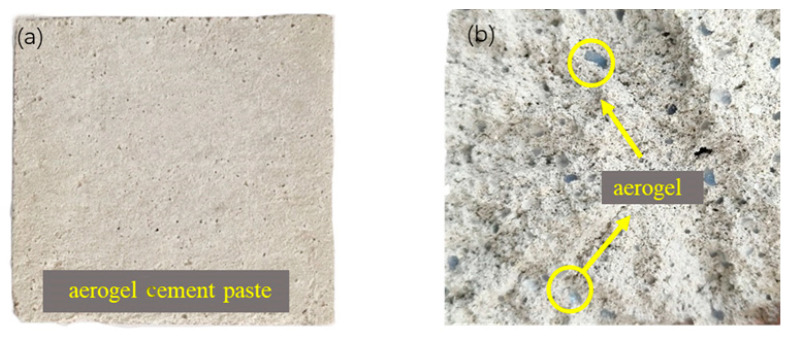
The ACP morphology before the simulated tunnel fire: (**a**) surface morphology; (**b**) section morphology.

The macroscopic morphology of the ACP before the simulated tunnel fire is shown in Figure 5. As shown in Figure 5a, the surface morphology of the ACP before the fire was grayish-white and some small holes were formed due to shrinkage of the cement slurry. In Figure 5b, transparent or blue aerogel particles of 0–2 mm were distributed inside ACP in good condition.

**Figure 6:**
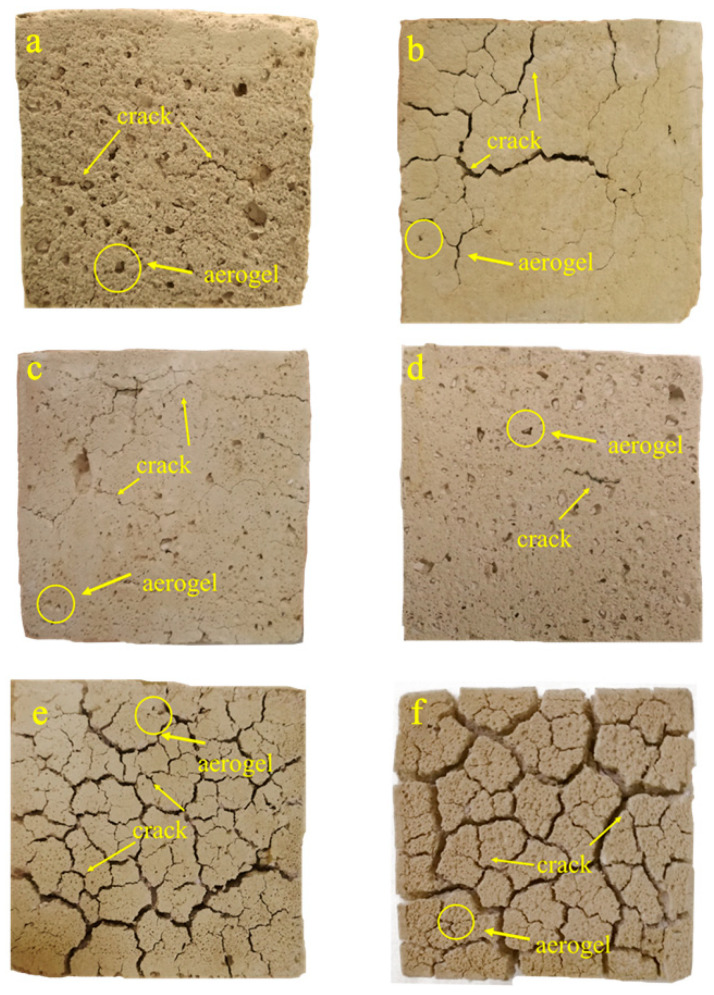
The ACP morphology after the simulated tunnel fire under different early curing regimes: (**a**) ACP morphology under 10 C; (**b**) ACP morphology under 20 C; (**c**) ACP morphology under 30 C; (**d**) ACP morphology under 40 C; (**e**) ACP morphology under 50 C; (**f**) ACP morphology under 60 C.

Figure 6 shows the morphology of ACP under different early curing conditions after the simulated tunnel fire. In general, the ACP surface morphology was gray and yellow after the tunnel fire test. No large areas of spalling occurred; although cracks generated, indicating the high stability of ACP in a tunnel fire. In addition, the cracks in the ACP under different early curing temperatures were not the same. In Figure 6a, the ACP under 10 °C early curing had two tiny cracks with loose texture after the tunnel fire. In Figure 6b, the ACP had a large main crack with a small number of tiny cracks around it. In Figure 6c,d, with the increase of early curing temperature (20–40 °C), the cracks gradually decreased, with only a small number of pores appearing. The ACP under 40 °C early curing temperature was the best preserved and showed the minimum number of pores. In Figure 6e,f, a large number of cracks and connected pores formed in the ACP under the early curing temperature of 50 and 60 °C. This indicated that severe damage was caused by the tunnel fire and the tunnel fire resistance of SCC coated with ACP could be reduced when the early curing temperature is increased to 50 °C.

It is evident that the aerogels on the surface shrank greatly and had suffered damage visible after the tunnel fire. Some scholars [28] pointed out that in order to make better use of the low thermal conductivity of aerogel, it can be used in combination with other materials to enhance its fire resistance.

### 3.3. Mass Loss of ACP

**Figure 7:**
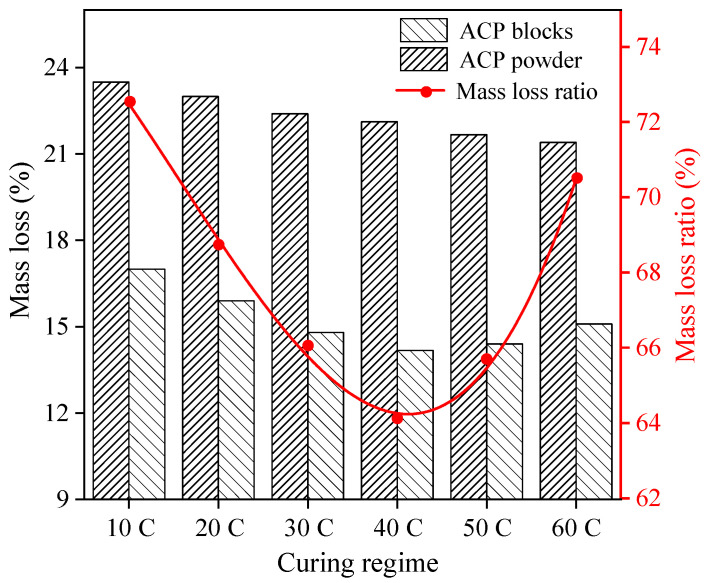
Mass loss of the ACP blocks and ACP powder and their ratio under different early curing regimes.

Figure 7 shows the mass loss of the ACP specimen and the 10 mg ACP powder before and after the tunnel fire and the ratio between them (mass loss ratio). The mass loss of the ACP powder was determined by the thermal conductivity of hydration products when neglecting the specimen microstructure. The mass loss of the ACP specimen and the mass loss ratio of the ACP reflected the combined effect of the microstructure and the thermal conductivity of hydration products. In Figure 7, the mass loss of the ACP powders gradually reduced with early curing temperatures. The minimum mass loss of the ACP powders was under 60 °C early curing (21.4%). This suggests that early high-temperature curing could make dense structure hydration products, which are beneficial in improving the fire resistance of ACP hydration products.

As shown in Figure 7, the mass loss of the ACP specimen or the mass loss ratio of ACP decreased at first and then increased with the increase of early curing temperatures. The ACP specimen with 40 °C early curing showed a minimum mass loss ratio with a value of 63%. Excessively high early curing temperatures led to an uneven microstructure and internal cracks, which had a negative impact on the fire resistance of the ACP specimen. In conclusion, with respect to the ACP microstructure, 40 °C early curing temperature was optimal to obtain a better tunnel fire resistance and heat insulation performance of the ACP.

### 3.4. The Residual Compressive Strength of SCC Coated with APC

**Figure 8:**
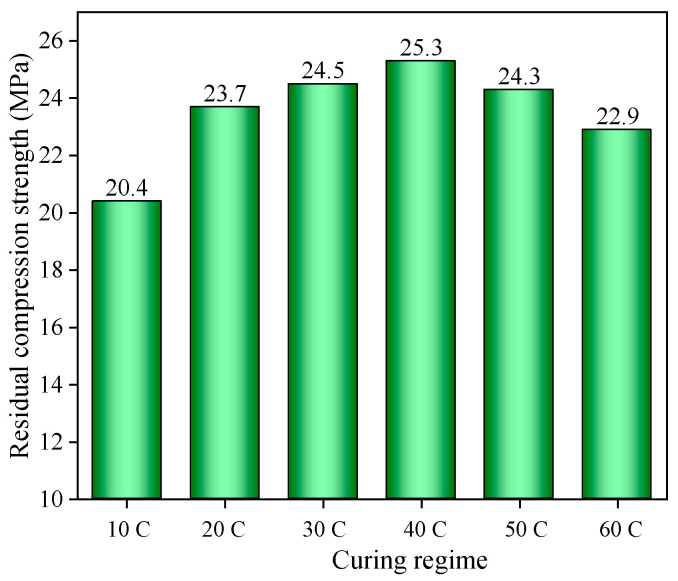
Residual compressive strength of SCC coated with ACP under different early curing regimes.

The residual compressive strength of SCC coated with ACP under different early curing regimes is shown in Figure 8. It can be seen that the residual compressive strength of the SCC gradually enhanced with the early curing temperature when the temperature was 10–40 °C. Higher early curing temperature accelerated the hydration reaction of ACP. More calcium hydroxide can react with silica fume and fly ash to generate stable C-S-H gel, which can raise the tunnel fire resistance and heat insulation performance of the ACP. However, the residual compressive strength of the SCC decreased with the early curing temperature when the temperature was 40–60 °C. The residual compressive strength of the SCC under 60 °C early curing reduced nearly 10% compared with that under 40 °C early curing. The effect of curing temperature on the performance of specimens as reported in other studies is shown in Table 8. When the curing temperature was greater than 90 °C, the compressive strength and flexural strength decreased. When the curing temperature was 20–80 °C, the compressive strength of the specimen increased at first and then decreased. The curing temperature corresponding to higher performance was approximately 40–50 °C, which was similar to the experimental results.

In addition, the compressive strength of SCC before the tunnel fire was also measured with the value of 42.3 MPa. It was found that the compressive strength of the SCC after the tunnel fire was only 50–60% of the initial compressive strength, in spite of the protection of 10 mm ACP fireproof coating. Therefore, it is suggested that the coating thickness of ACP be appropriately increased in order to improve the residual compressive strength of SCC after a tunnel fire in actual engineering.

### 3.5. SEM Analysis of ACP before and after Tunnel Fire

**Figure 9:**
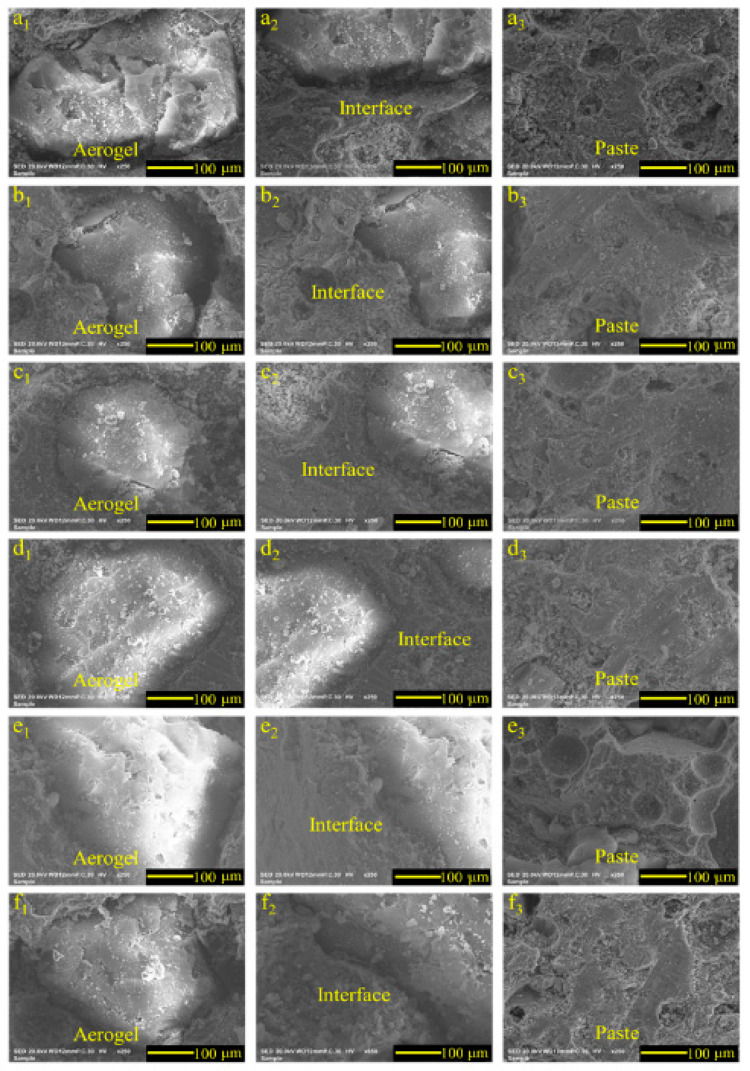
SEM images of ACP under different early curing regimes before the simulated tunnel fire: (**a_1_**) aerogel under 10 C; (**a_2_**) interface under 10 C; (**a_3_**) paste under 10 C; (**b_1_**) aerogel under 20 C; (**b_2_**) interface under 20 C; (**b_3_**) paste under 20 C; (**c_1_**) aerogel under 30 C; (**c_2_**) interface under 30 C; (**c_3_**) paste under 30 C; (**d_1_**) aerogel under 40 C; (**d_2_**) interface under 40 C; (**d_3_**) paste under 40 C; (**e_1_**) aerogel under 50 C; (**e_2_**) interface under 50 C; (**e_3_**) paste under 50 C; (**f_1_**) aerogel under 60 C; (**f_2_**) interface under 60 C; (**f_3_**) paste under 60 C.

SEM images of ACP under different early curing conditions before the tunnel fire are shown in Figure 9. It can be seen that there was a pore (nearly 1–5 μm) between the aerogel and paste, which was caused by shrinkage of the paste during curing. Ng et al. [32] pointed out that these pores are conducive to water transport in the curing process, which is better for ACP curing. In Figure 9, the ACP specimen under 40 °C early curing had the smallest micropores. There were more pores in the ACP specimen under 10 °C early curing than that under 40 °C early curing, which was due to the slow hydration reaction and uneven paste when the early curing temperature was 10 °C. Appropriately raising the early curing temperature was conducive to the densification of the ACP microstructure. In contrast, the ACP specimen under 50 °C and 60 °C early curing had more rough pores than those under 30 °C and 40 °C early curing. In addition, internal cracks caused by early fast curing can be seen in the ACP specimen under 60 °C early curing. The pores produced in early curing could accelerate damage of the ACP specimen during a tunnel fire.

**Figure 10:**
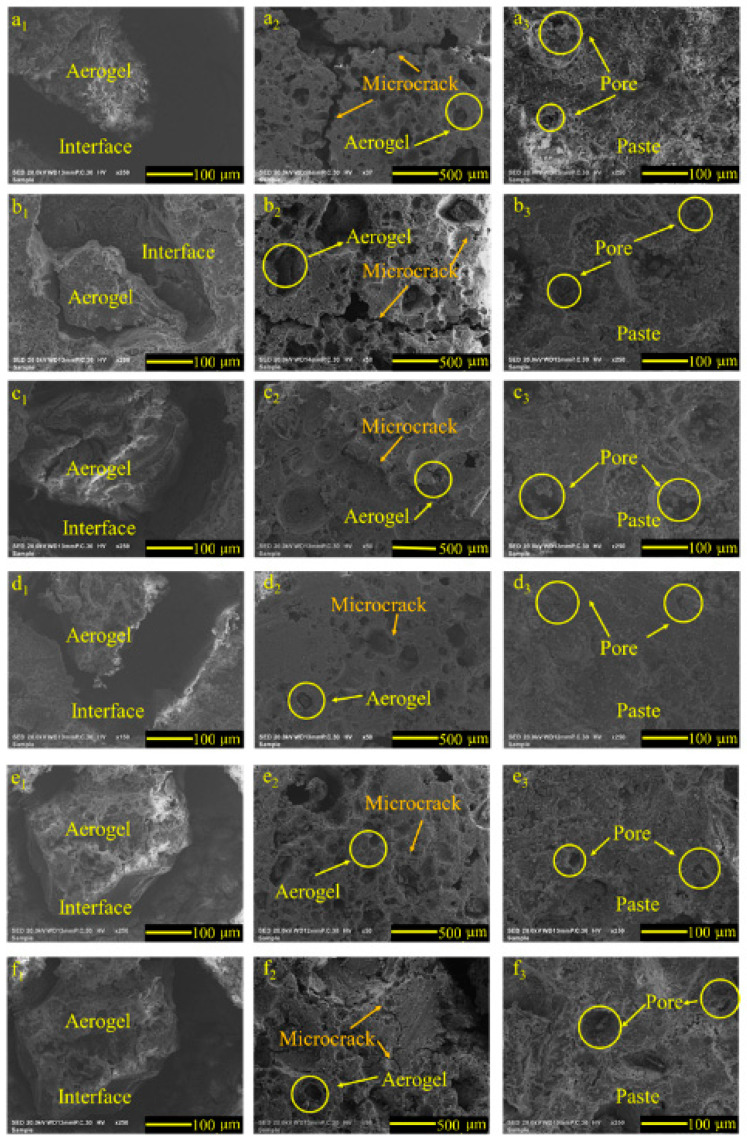
SEM images of ACP under different early curing regimes after the simulated tunnel fire: (**a_1_**) interface under 10 C; (**a_2_**) aerogel under 10 C; (**a_3_**) paste under 10 C; (**b_1_**) interface under 20 C; (**b_2_**) aerogel under 20 C; (**b_3_**) paste under 20 C; (**c_1_**) interface under 30 C; (**c_2_**) aerogel under 30 C; (**c_3_**) paste under 30 C; (**d_1_**) interface under 40 C; (**d_2_**) aerogel under 40 C; (**d_3_**) paste under 40 C; (**e_1_**) interface under 50 C; (**e_2_**) aerogel under 50 C; (**e_3_**) paste under 50 C; (**f_1_**) interface under 60 C; (**f_2_**) aerogel under 60 C; (**f_3_**) paste under 60 C.

Figure 10 shows the SEM images of ACP under different early curing conditions after the tunnel fire. It can be seen from the figure that the aerogel shrank after the tunnel fire, and there were huge cracks between the paste and aerogel, especially for the ACP specimen under 10 °C and 20 °C early curing. The microcracks of the ACP specimen under 40 °C early curing were the shortest.

Too high or too low early curing temperatures increased the porosity of the ACP specimen, which can reduce the thermal conductivity and enhance the thermal insulation ability. However, it also exerted adverse effects on the ACP microstructure [29,30,31], accelerating the formation and expansion of microcracks during the tunnel fire, which reduced the fire resistance and heat insulation performance of ACP. With respect to the thermal conductivity and microstructure, ACP under 40 °C early curing had the best tunnel fire resistance and heat insulation performance.

### 3.6. SEM Analysis of SCC Coated with ACP after Tunnel Fire

**Figure 11:**
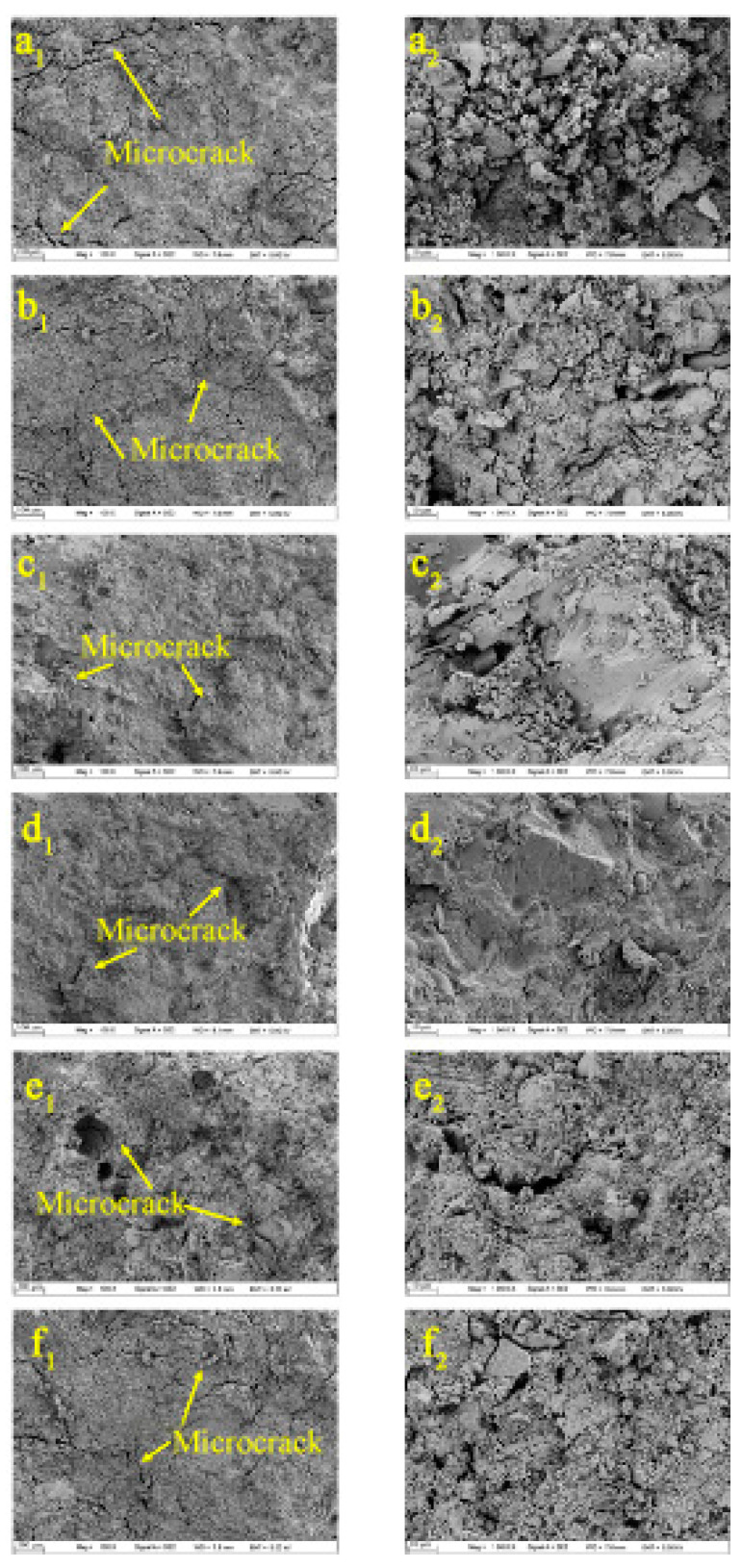
SEM images of SCC coated with ACP under different early curing regimes after the simulated tunnel fire: (**a_1_**) aggregate under 10 C; (**a_2_**) mortar under 10 C; (**b_1_**) aggregate under 20 C; (**b_2_**) mortar under 20 C; (**c_1_**) aggregate under 30 C; (**c_2_**) mortar under 30 C; (**d_1_**) aggregate under 40 C; (**d_2_**) mortar under 40 C; (**e_1_**) aggregate under 50 C; (**e_2_**) mortar under 50 C; (**f_1_**) aggregate under 60 C; (**f_2_**) mortar under 60 C.

Figure 11 shows the SEM images of SCC with different early curing after the simulated tunnel fire test. In general, microcracks occurred in SCC, indicating that SCC coated with ACP still suffered damage from the tunnel fire. By comparing the SEM pictures of SCC under different early curing conditions, it can be seen that the width of microcracks in SCC decreased gradually at first and then increased with the early curing temperature. The microcrack width of SCC with 40 °C early curing was the least; it showed the best tunnel fire resistance, which is consistent with the results of residual compressive strength. The SCC under 10 °C early curing showed the largest and longest microcracks, suggesting that it is better to raise the early curing temperature in practical engineering.

## 4. Conclusions

The following conclusions can be drawn:

(1) The compressive strength and flexural strength of ACP increased at first and then decreased later with the increase of early curing temperature. The highest compressive strength and flexural strength of ACP occurred in the specimen under 40 °C early curing.
(2) For the ACP specimen, the aerogels on the surface shrank greatly and cracks generated after the tunnel fire, although no large area of spalling occurred. The ACP under 40 °C early curing exhibited the minimum number of cracks and mass loss after the tunnel fire.
(3) The residual compressive strength of SCC coated with 10 mm ACP after the tunnel fire was only 50–60% of the initial compressive strength. The residual compressive strength of SCC increased at first as the early curing temperature of the ACP increased up to 40 °C and then decreased when the temperature of the ACP increased from 40 °C to 60 °C.
(4) Too high or too low early curing temperature reduced the thermal conductivity of ACP but accelerated the formation and expansion of microcracks during the tunnel fire.
(5) With respect to the thermal conductivity and microstructure, ACP under 40 °C early curing had the best tunnel fire resistance and heat insulation performance. In addition, the SCC coated with ACP under 40 °C early curing showed the best tunnel fire resistance.

## Figures and Tables

**Table 1:** Chemical composition of cement, fly ash, and silica fume (% by mass).

Binding Material	SiO_2_	Al_2_O_3_	Fe_2_O_3_	TiO_2_	SO_3_	CaO	Na_2_O	MgO	K_2_O	Other
Cement	12.54	4.41	3.41	0.59	1.96	72.97	0.26	0.21	0.90	2.75
Fly ash	37.96	24.35	12.06	1.97	0.95	14.83	2.27	1.03	1.86	2.72
Silica fume	94.30	0.04	–	–	0.08	1.21	–	0.08	0.01	4.28

**Table 2:** Performance parameters of water reducer.

Water Reduction Rate (%)	Chloride Ion Content (%)	Alkali Content (%)	Water Content (%)	Solid Content (%)	pH
25	0.038	≤0.2	<3	40 ± 2	8.3

**Table 3:** Performance parameters of air-entraining agent.

Ratio of Bleeding Rate (%)	Gas Content (%)	Solid Content (%)	Corrosion of Reinforcement	pH
49.3	5.3	15	No	8.2

**Table 4:** Physical characteristics of SiO_2_ aerogel.

Particle Size (mm)	Porosity (%)	Density (kg/m^3^)	Thermal Conductivity (W/m·K)
0–2	> 90	100	0.020

**Table 5:** Mix proportion of ACP (kg/m^3^).

Cement	Water	Aerogel	Silica Fume	Fly Ash	Air-Entraining Agent	Water Reducer
324	180	65	36	90	4.5	4.5

**Table 6:** Mix proportion of C40 SCC (kg/m^3^).

Cement	Fly Ash	Silica Fume	Slag	Water	Fine Aggregate	Coarse Aggregate	Water Reducer
335	111.7	55.8	55.8	173.1	814	858	1.7

**Table 7:** The different curing regimes.

Curing Regime	Early Curing (RH > 95%)	Wet Curing Time (13–24 °C, 70 ± 15% RH)	Dry Curing Time (13–24 °C, 70 ± 15% RH)
10 C	10 °C, 1 days	14 days	14 days
20 C	20 °C, 1 days	14 days	14 days
30 C	30 °C, 1 days	14 days	14 days
40 C	40 °C, 1 days	14 days	14 days
50 C	50 °C, 1 days	14 days	14 days
60 C	60 °C, 1 days	14 days	14 days

**Table 8:** Effect of curing temperature on the performance of specimens.

Reference	Temperature (°C)	Compressive Strength (MPa)	Flexural Strength (MPa)	Permeability (mm)	Thermal Conductivity (W/m·K)
[2]	60–70	↓	–	↑	–
[21]	80–120	↓	↓	–	↓
[25]	15–70	↓	–	–	–
[26]	20–90	↑first, ↓ after 50 °C	–	–	–
[29]	60–100	↓	↓	–	–
[30]	20–50	↑	–	–	–
[31]	20–60	↑first, ↓ after 40 °C	–	–	–

## Data Availability

The data presented in this study are available upon request from the corresponding author.
